# The effect of wide resection margin in patients with intrahepatic cholangiocarcinoma: A single-center experience

**DOI:** 10.1097/MD.0000000000004133

**Published:** 2016-07-18

**Authors:** Ka Wing Ma, Tan To Cheung, Wong Hoi She, Kenneth S.H. Chok, Albert Chi Yan Chan, Irene Oi Lin Ng, See Ching Chan, Chung Mau Lo

**Affiliations:** a Department of Surgery, The University of Hong Kong; b State Key Laboratory for Liver Research, The University of Hong Kong; c Department of Pathology, The University of Hong Kong, Hong Kong, China.

**Keywords:** hepatectomy, ICC, liver resection, long-term outcome, survival analysis

## Abstract

**Introduction:**

Prognosis of intrahepatic cholangiocarcinoma (ICC) remained poor despite the multitude advancement of medical care. Resection margin status is one of the few modifiable factors that a surgeon could possibly manipulate to alter the disease outcome. However, the significance of margin status and margin width is still controversial. This study serves to further elucidate the role of them.

**Method:**

This is a retrospective cohort from the Queen Mary Hospital, The University of Hong Kong. Consecutive patients diagnosed to have ICC and with surgical resection performed in curative intent were retrieved, while patients with cholangiohepatocellular carcinoma, Klaskin tumor, tumor of extrahepatic bile duct, and uncertain tumor pathology were excluded.

**Results:**

From 1991 to 2013, there were 107 patients underwent hepatectomy for ICC. Gender predilection was not observed with 58 males and 49 females, median age of the patients was 61. The median tumor size was 6 cm and most of them (43%) were moderately differentiated adenocarcinoma. Clear resection margin were achieved in 95 patients (88.8%) and the median margin width was 0.5 cm. The hospital length of stay and operative mortality were 11 days and 3%, respectively. The disease-free survival and overall survival were 17.5 and 25.1 months, respectively. Multivariate analysis showed that margin width was an independent factor associated with disease-free survival (*P* = 0.015, 95% confidence interval [CI] 0.4–0.9). Subgroup analysis in patients with solitary tumor showed that margin width is an independent factor affecting overall survival (*P* = 0.048; odds ratio: 0.577; 95% CI: 0.334–0.996). Discriminant analysis showed that the overall survival increased from 36 to 185 months when margin width was >0.9 cm (*P* = 0.025) in patients with solitary tumor.

**Conclusion:**

Aggressive resection to achieve resection margin of at least 1 cm maximizes chance of cure in patients with early ICC.

## 1 Introduction

Cholangiocarcinoma is an uncommon malignancy, which accounts for <2% of all human cancers.^[1]^ While intrahepatic cholangiocarcinoma (ICC) contributes to around 20% to 25% of all cholangiocarcinomas, it ranks the second most common primary cancer of the liver following hepatocellular carcinoma.^[2]^ Pathologically, it can be classified into 3 types, namely, mass-forming, peri-ductal infiltrative, and intraductal growth.^[3]^ Unlike the least prevalent intraductal growth type, ICC is generally associated with poor prognosis; this is partly due to its aggressive tumor biology^[4]^ and late presentation secondary to a relatively asymptomatic intrahepatic tumor.^[5–7]^ Only 10% to 20% of the ICCs are deemed resectable at the time of presentation^[8]^ and the median survival ranges from 6 to 9 months for patients with unresectable disease.^[9–11]^

Surgical resection remains the only hope of cure in patients with ICC, and the 5-year survival after surgical resection is around 25% to 35%.^[12]^ Despite the multitude advancement in various aspects of hepatic operation, there has been little breakthrough concerning the survival of patients with ICC in the last decade.^[7,13]^ Studies had looked into the factors that might potentially influence treatment outcomes, these included tumor size, tumor multifocality, vascular invasion, lymph node status, and resection margin status.^[5–7,14–23]^ Among all of these factors, only resection margin and the width of clear resection margin were technically modifiable by the operating surgeon. The practice of aggressive hepatectomy to strive for a wider resection margin in order to improve survival has been controversial. The findings of a recently published multicenter study by the French Association Francophone de Chirurgie–Intrahepatic Cholangiocarcinoma (AFC-IHCC)-2009 Study Group advocated a “wider the better” concept in selected group of patients.^[22]^ The generalizability of this result to oriental patients has yet to be confirmed, furthermore, a number of heterogeneities exemplified by multicenter study could be mitigated by a well-designed, single-center series with reasonable sample size. Therefore, we perform this study to the share our experience in managing ICC and to further explore the association between resection margin and patient survivals.

## 2 Patients and methods

### 2.1 Patient selection

Patients diagnosed to have ICC and with subsequent hepatectomy performed with curative intent in the period between 1991 and 2013 were extracted from a prospectively maintained database in Queen Mary Hospital, The University of Hong Kong medical center. Patients with cholangiohepatocellular carcinoma, Klaskin tumor, and cholangiocarcinoma arising from extrahepatic biliary tract or suspected metastatic adenocarcinoma of liver were excluded from analysis. All patients included in our series had at least macroscopic (R0 or R1) curative resection. Diagnosis was confirmed by dedicated hepatobiliary pathologists. ICC by definition should arise from the periphery of biliary system, that is, second-order bile duct branching onward. In addition to standard microscopy and staining, immunohistochemical tests including cytokeratin-7, cytokeratin-20, and thyroid transcription factor-1 would be performed whenever necessary.

### 2.2 Preoperative assessments

Patients with working diagnosis of ICC would undergo a series of biochemical and radiological assessments before embarking on hepatectomy. Complete blood count, liver, renal function tests, and clotting profile were checked as routine. Hepatitis serology, baseline level of carcinoembryonic antigen (CEA), and Child-Pugh scoring were documented. All patients would undergo indocyanine green (ICG) clearance test, ICG retention of 18% and 22% at 15 min after injection were regarded as the cut-off level for major and minor hepatic resection, respectively. Radiologically, a contrasted computed tomography (CT) with or without 18-F 2-fluoro-2-deoxy-d-glucose positron emitted tomography was performed to define local anatomy and presence of systemic involvement. A CT volumetric study would be required for patients requiring major hepatectomy (resection of 3 or more Couinaud segments). The minimum ratio of future liver remnant volume versus estimated standard liver volume was 30% for noncirrhotic livers.^[24,25]^ In case of marginal liver volume, percutaneous image guided portal vein embolization or associating liver partition with portal vein ligation for staged hepatectomy could be considered on individual basis in a multidisciplinary meeting.

### 2.3 Definitions

The nomenclature of liver anatomy and resection follows that of the International Hepato-Pancreato-Biliary Association (Brisbane 2000) consensus statement.^[26]^ Hospital mortality was defined as any mortality happened in the first 30 days after the operation and operative mortality was defined as any mortality happened within the first 90 days after operation. Clear resection margin (R0) was defined as the distance between the nontumorous tissue and cancer cell >1 mm. R1 resection referred to resection margin touching inked tumor, while R2 resection means gross residual disease, which was an exclusion criteria in this study. Postoperative complications were classified according to Clavien-Dindo description.^[27]^ All cancer staging follows that of the Union for International Cancer Control/Tumor-Node-Metastasis (TNM) 7th edition.^[28]^

### 2.4 Surgical techniques

The technical details of hepatectomy in our center had been described in another report.^[24]^ Operation started with either midline incision, right subcostal with midline extension, or bilateral subcostal with midline extension depending on the location of tumor and anticipated magnitude of hepatectomy. Routine intraoperative ultrasonography (IOUS) was performed to look for any vascular invasion; presence of tumor in the contralateral lobe precludes curative resection. Transection plane was defined by color demarcation after temporary inflow occlusion and IOUS vascular mapping. Liver transection was then started with the use of Cavitron ultrasonic dissector in either conventional or anterior approach depending on the location and size of tumor. Frozen section for bile duct resection margin was not routinely done unless tumor extension was suspected. Vasculobiliary resection and reconstruction was contemplated whenever necessary to achieve R0 resection. Caudate lobe resection was not routinely included unless the tumor was centrally locating. Central venous pressure was kept at around 5 mm Hg during parenchymal transection. Monopolar, bipolar diathermy, argon beam coagulation, metal clips, fine suture placation, and various commercial hemostatic products would be applied for hemostasis. Intermittent Pringle maneuver or total vascular exclusion was applied only when excessive bleeding encountered. Bile leakage was checked by cannulation of cystic duct with a 5-french Argyle catheter followed by injection of methylene blue solution. Drain was not placed unless collection was anticipated. Patient could be discharged home around 1 to 2 weeks depending on individual progress of recovery.

### 2.5 Follow-up and surveillance

All patients were followed up at outpatient clinic within 2 weeks after discharge home and then every quarter-yearly for the first year and half-yearly for the second year onward according to individual progress. Routine blood tests including liver function and CEA were checked before every follow-up and cross-sectional imaging was arranged every half-yearly or earlier whenever necessary. Disease recurrence was declared if contrast CT shows features of local recurrence or distant metastasis.

### 2.6 Statistics

Continuous variables were described as median with range included in bracket. Categorical variables were analyzed using Chi-squared test or Fisher exact test where appropriate. Parametric variables were analyzed using Mann–Whitney *U* test or *t* test where appropriate. Disease-free and overall survivals were calculated from the day of discharge to the day of disease recurrence or census, that is, death or last follow up, using Kaplan–Meier method. Survivals between groups were compared using Log-rank test. Variables associated with survival with *P* < 0.1 in univariate analysis were put into multivariate analysis in order to identify the independent factors. *P* value of <0.05 was considered statistically significant. Discriminant analyses were performed to identify cut-off values of margin width that affect survival. Statistical Product and Service Solutions version 20 was used for all statistical analysis.

### 2.7 Ethics and declarations

This study does not require ethics board review according to local guidelines. All patient identities and clinical information were kept confidential. The authors declare no known benefit from this article.

## 3 Results

From 1991 to 2013, there were 107 consecutive patients received intended curative hepatectomy for ICC and were included for analysis. Gender predilection was not observed, with male patients being slightly more common than their female counterparts (58 males and 49 females). The median age was 61 (25–79) years old and the follow-up time was 21.4 months (0.2–276.3). Hepatitis B carrier state was confirmed in 33.6% of our patients. Pathological changes compatible with of underlying recurrent pyogenic cholangitis (RPC) was noted in 17 patients (15.9%). The hospital length of stay and hospital mortality of our series were 11 days and 3%, respectively (Table 1). The 5-year disease-free and overall survivals of this series were 39% and 36.7%, respectively. There were 7 (6.5%) patients died within 90 days of operation. Postoperative complications occurred in 34 patients (31.8%), with grade IIIA, IIIB, IV, and V complication occurred in 14, 3, 2, and 8 patients, respectively.

**Table 1:**
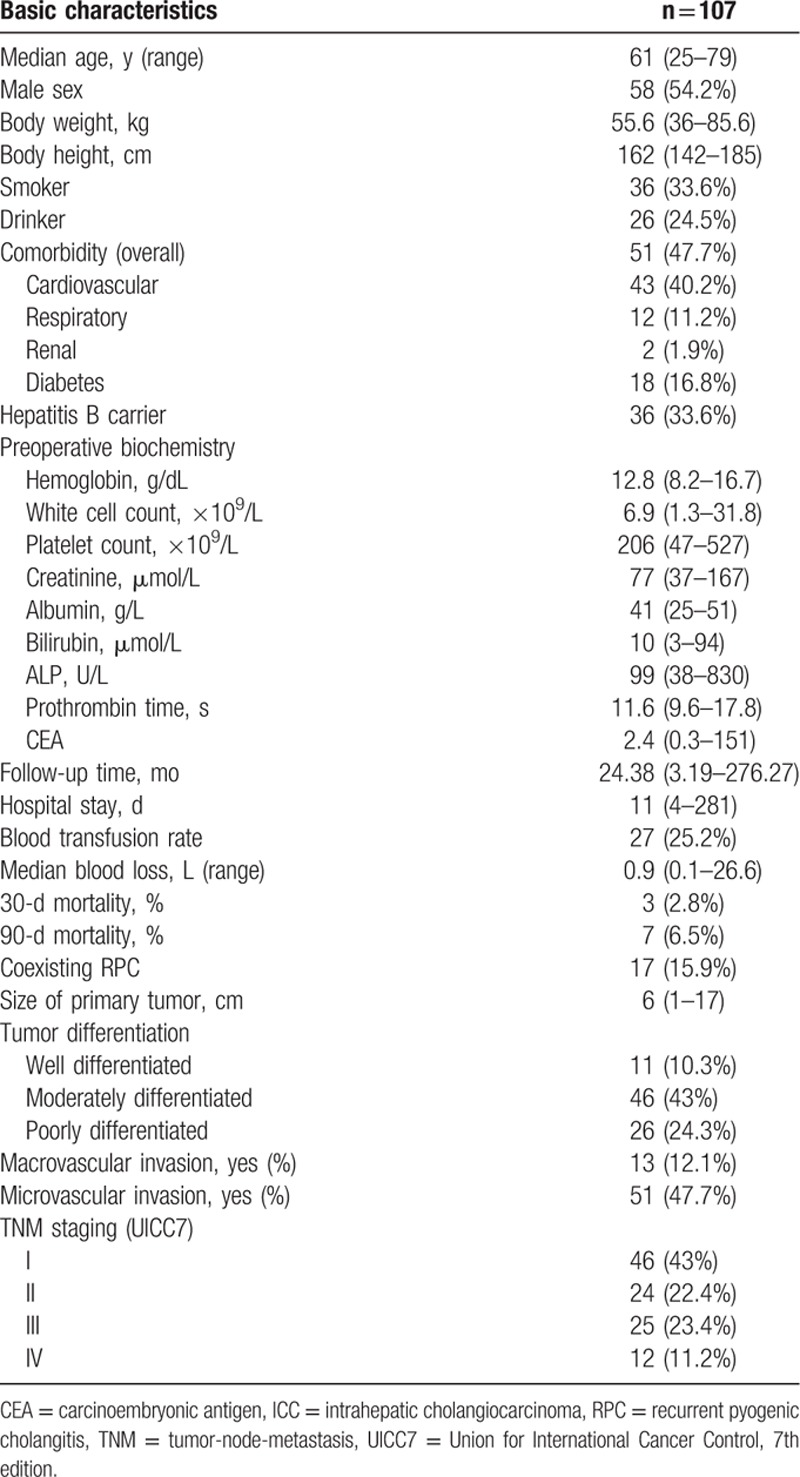
Clinicopathological characteristics of 107 patients with ICC.

Majority of our patients required major hepatectomy (defined as more than 3 segmental resection) for tumor resection, and right trisectionectomy was the most commonly performed surgical procedure (Table 2). Added procedure was required in 21 patients (19.5%), bile duct resection was performed in 15 patients, other 5 had portal vein, inferior vena cava, or hepatic artery resection and reconstruction, 1 patient had intraoperative radiofrequency ablation of tumor performed.

**Table 2:**
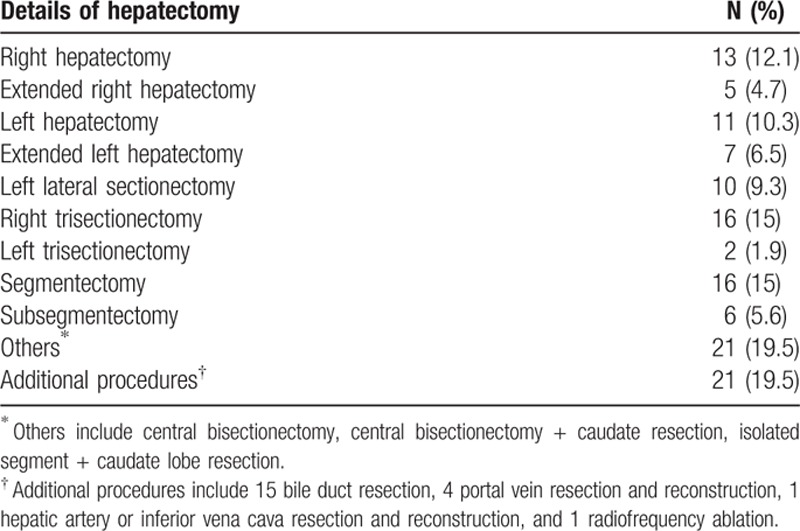
Frequency of various surgical procedures performed.

**Figure 1:**
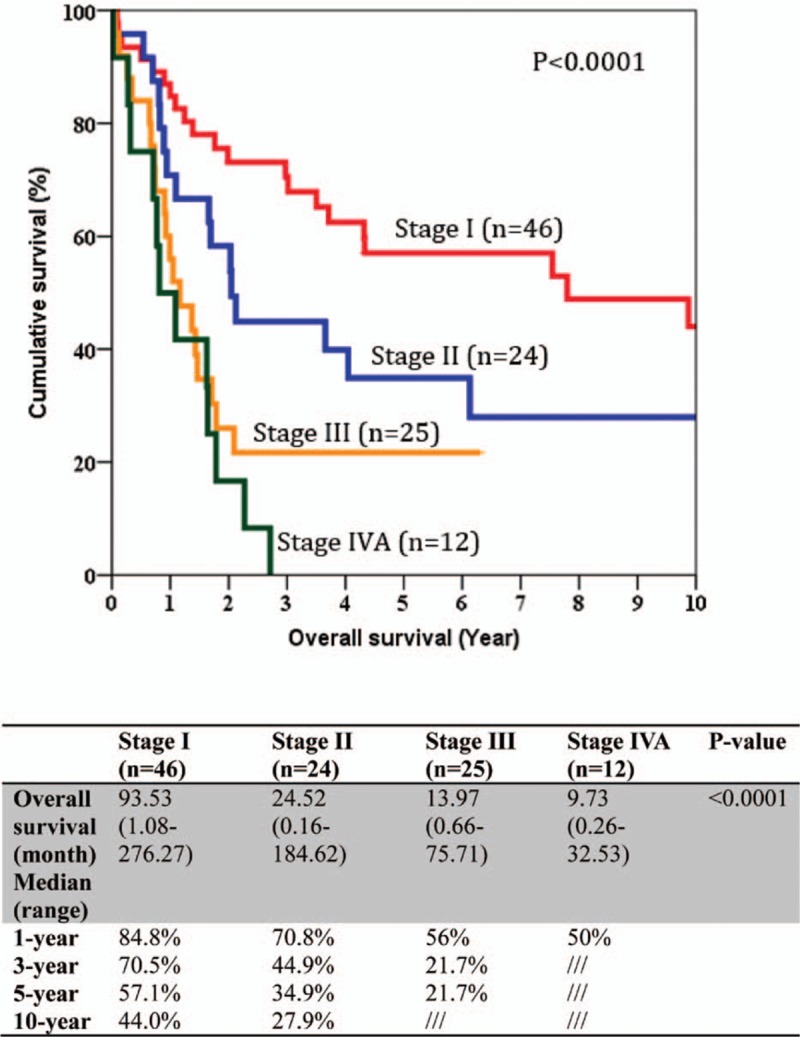
Stage-specific overall survival of patient with intrahepatic cholangiocarcinoma after operation.

**Figure 2:**
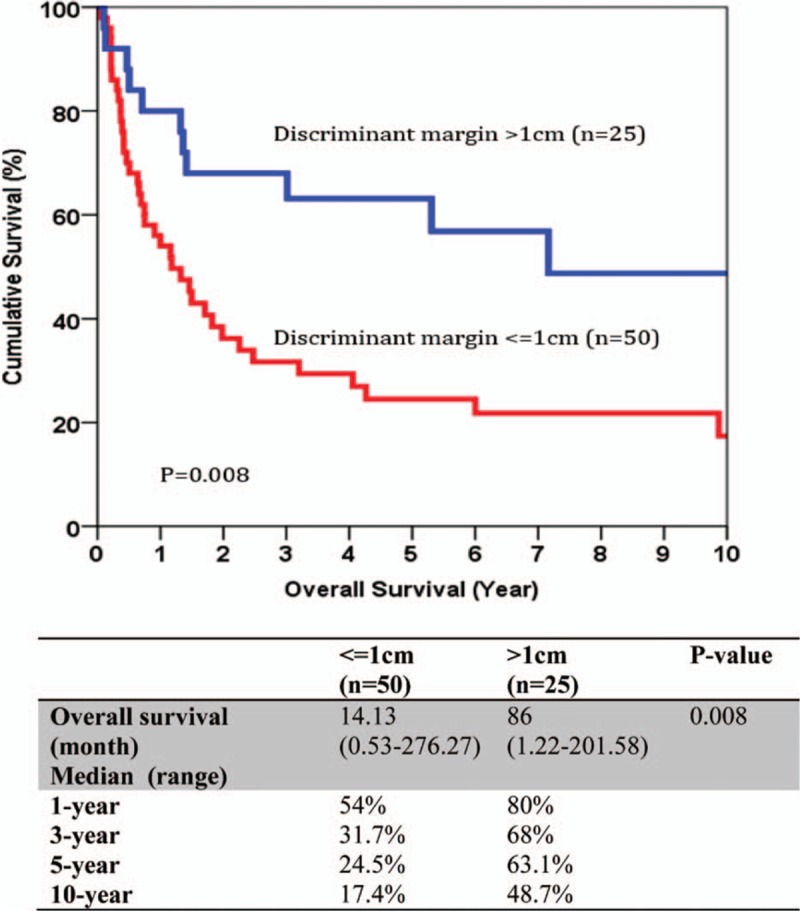
Discriminant analysis for margin width cut-off value for disease-free survival.

**Figure 3:**
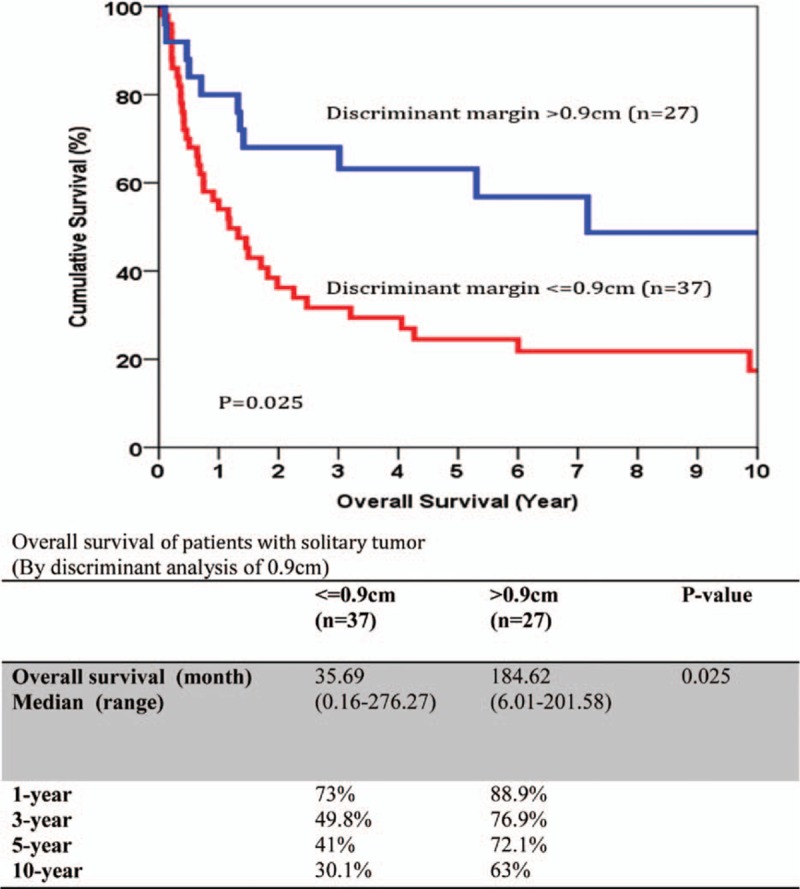
Discriminant analysis for margin width cut-off value for overall survival for patients with solitary intrahepatic cholangiocarcinoma.

The median size of the primary tumor was 6 cm (1–17 cm) and most of the tumors were moderately differentiated adenocarcinoma. For the TNM staging, there were 43%, 22%, 23%, and 12% of the patients belonged to stage I, II, III, and IV, respectively, and the overall survival correlate well with TNM staging (Fig. 1). All patients had macroscopic clear resection with only 12 patients had microscopic margin involvement according to definition. R0 resection was achieved in 95 patients (88.8%), and the rate of R0 resection seemed not to be affected by the operation time, magnitude of hepatectomy, need of additional procedure, tumor size, presence of vascular invasion, and TNM staging (Table 3). The median margin width was 0.5 cm. The median time to first recurrence was 9.7 months (0.53–160.6). Tumor recurrence documented in 58% in our series, and most of them had both intrahepatic and extrahepatic recurrences (22.4%). A smaller percentage of patients had intrahepatic recurrence only (15%). The disease-free and overall survivals were 17.5 months (0.5–276.3) and 25.1 months (0.2–276.3), respectively. Univariate analysis was performed for a number of clinicopathological factors (Table 4) and there were 6 factors identified to be associated with disease-free survival of the patients, namely primary tumor size (*P* = 0.026), postoperative surgical complication (*P* = 0.006), multifocality (*P* < 0.0001), width of resection margin (*P* = 0.015), and TNM staging (*P* = 0.015). After multivariate analysis, only postoperative surgical complication (*P* = 0.032, 95% confidence interval [CI]: 1.1–3.2), TNM staging (*P* = 0.001), and width of resection margin (*P* = 0.015, 95% CI: 0.4–0.9) stand out as the independent factors affecting disease-free survival. In order to find out the minimal resection margin required to obtain an improved result, a discriminant analysis was performed and it was found that the disease-free survival increased from 14.1 to 86 months when width of resection margin is more than 1 cm (*P* = 0.008) (Fig. 2). Concerning the overall survival, 6 factors were shown to be associated after univariate analysis, including preoperative intraoperative blood loss (*P* = 0.014), surgical complication (*P* < 0.0001), multifocality (*P* < 0.0001), width of resection margin (*P* = 0.014), and TNM staging (*P* < 0.0001). Although multivariate analysis showed that only postoperative complication (*P* = 0.0004, odds ratio [OR]: 2.87; 95% CI: 1.649–4), and TNM staging (*P* < 0.05) were the 2 independent factors affecting overall survival (Table 5). Subgroup analysis was performed in patients with solitary tumor and showed that width of resection margin is an independent factor affecting overall survival (*P* = 0.048; OR: 0.577; 95% CI: 0.334–0.996) (Table 6). Discriminant analysis was performed in this subgroup and found that if resection margin more than 0.9 cm, the median survival would be increased from 35.7 to 184.6 months (*P* = 0.025) (Fig. 3).

**Table 3:**
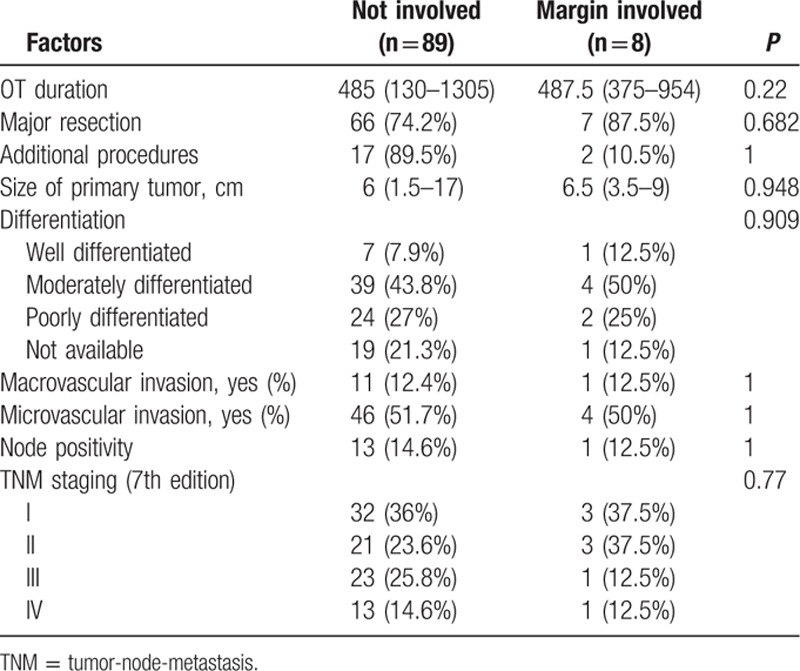
Factors affecting achievement of R0 resection.

**Table 4:**
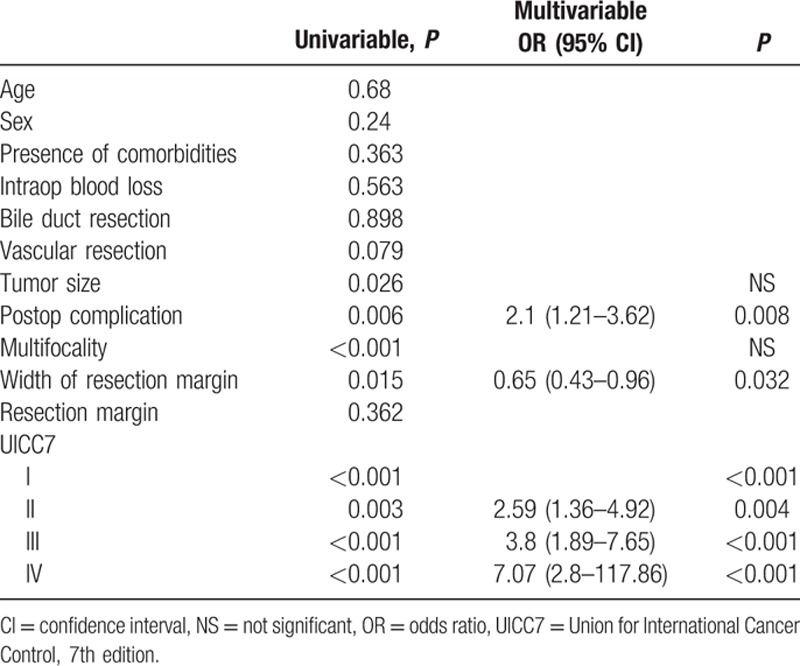
Univariate and multivariate analyses of various factors against disease-free survival.

**Table 5:**
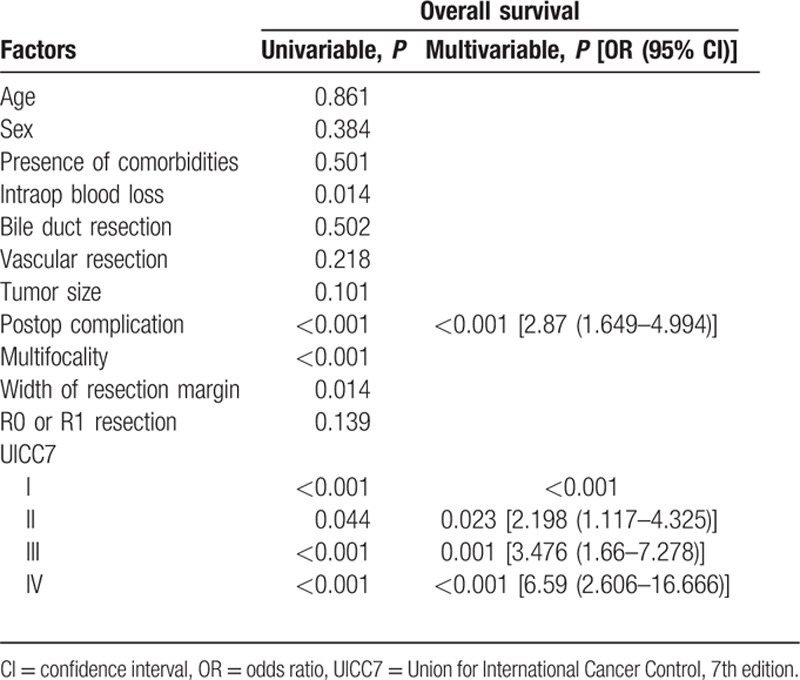
Univariate and multivariate analyses of various factors against overall survival.

**Table 6:**
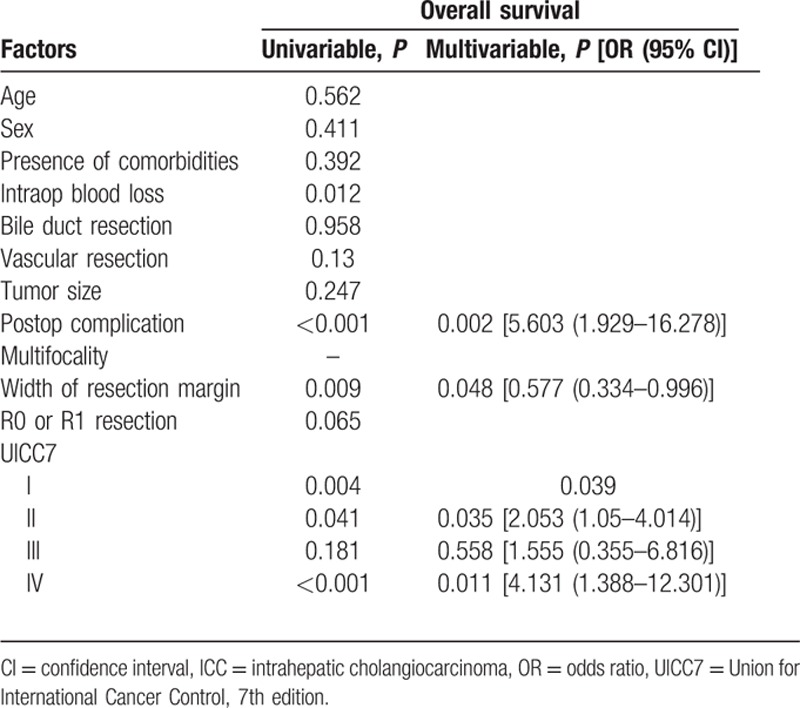
Factors associated with overall survival (subgroup analysis of patients with solitary ICC).

## 4 Discussion

To our knowledge, this is the largest single-center retrospective series focusing on oncological outcome of resectable ICC. In this series, the 5-year overall survival for patients who received operation of curative intent was 36.7% and this figure was comparable to other major series in the literatures.^[21,29–31]^ Some known risk factors associated with ICC included hepatitis B and C carrier state, cirrhotic liver disease, *Opisthorchis viverrini* and *Clonorchis sinensis* infestation, RPC, and choledochal cyst present only in about 10% of our patients. Given the fact that symptoms were uncommon in early stage of disease, biochemical and radiological surveillance should be performed on regular basis in patients who possess these risk factors.

Microscopic clear margin status has been regarded as the ultimate objective in resection of ICC and it had been shown by a number of studies that R0 resection was associated with improved survival^[5,6,14–18,20–22,32]^ while a few suggested the contrary.^[7,33,34]^ Despite we were unable to demonstrate R0 resection has oncological benefit over R1 resection, probably as a result of small case number in the R1 group (95 vs 12 in R0 and R1, respectively), the authors were inclined to support that R0 resection margin should be achieved as much as possible for an adequate oncological clearance. To account for over 10% R1 resection rate in this series, regardless of the relatively “small tumor size” (median tumor size was 6 cm), the reasons were 3-fold; anatomically, some of the tumors situated at strategic area rendering on-bloc resection too risky to be performed, hence a vascular or bile duct margin was relentlessly left behind; morphologically, irregular tumor border of infiltrative or mixed type ICC could lead to false sense of margin adequacy from both tactile and IOUS examination; lastly, skipped lesions or small satellite nodule could still be missed with the finest loupe or ultrasound machine. The last 2 reasons set the stage for advocating a more aggressive resection margin especially for seemingly solitary tumor and node negative disease. Concerning adequate width of resection margin, few literatures addressed this controversial issue directly^[35–38]^ and we demonstrated that the wider the width of resection margin, the longer is the disease-free survival and overall survival in selected group of patients. Belghiti et al^[38]^ reviewed 30 patients who had ICC and hepatectomy for curative intent. In that article they concluded that resection margin had no impact on survival. However, that finding could not be over-generalized as all of their patients were TMN stage III or above and with around 40% of them had terminal disease, that is, TMN stage IV; therefore, the beneficial effect of wide resection margin might well be masked by the “too far gone” advanced disease status. From our multivariate analysis, we found that resection margin width was an independent factor for disease-free survival and discriminant analysis performed showed that a cut-off width of resection margin 1 cm is associated with improved disease-free survival. This finding serves 2 purposes; on one hand, this should be the minimal margin width that the operating surgeon should aim for when they are operating on patient with apparent early disease. On the other hand, ultimate margin with as per pathological assessment helps to prognosticate disease outcome and to select high-risk group for adjuvant therapies. Though we could not establish the similar relationship with overall survival due to a number of reasons, that is, small case number and disease-unrelated mortality, our findings concurred with the study published by Farges et al^[22]^ who suggested wide resection margin in a similar group of patients. All these results suggest that, aggressive surgery achieving a wide resection margin could improve survival in patients with early ICC, that is, solitary tumor.

The retrospective nature of this study contributes to its major limitation, possibility of selection bias, missing data, and problems of treatment heterogeneity throughout the years could not be completely evaded. On the hand, the effect of adjuvant chemotherapy, which could potentially influence survival outcome, was not investigated in the current study. Our series included consecutive patients for analyses; hence the chance of selection bias was minimized. A single-center series also limits interobserver variability and treatment heterogeneity in terms of perioperative management and operative techniques. While it deems neither practical nor ethical to perform a prospective randomized trial on the effective margin width, this retrospective series of reasonable sample size serves to shed some light on the surgical approach of patients with ICC.

## 5 Conclusion

ICC is an uncommon primary liver malignancy associated with poor prognosis even operation can be done with curative intent. Aggressive operative approach to obtain resection margin at least 1 cm can improve patients’ survival outcomes.
